# Supplementary material for: Frailty as a Predictor of Outcomes in Subarachnoid Hemorrhage: A Systematic Review and Meta-Analysis
Source: Brain Sci. 2023 Oct 23;13(10):1498. doi: 10.3390/brainsci13101498 (PMC10605612; doi:10.3390/brainsci13101498)
Supplement: Supplementary file 1 [file brainsci-13-01498-s001.zip › brainsci-2639620-supplementary.pdf]

Supplemental Materials

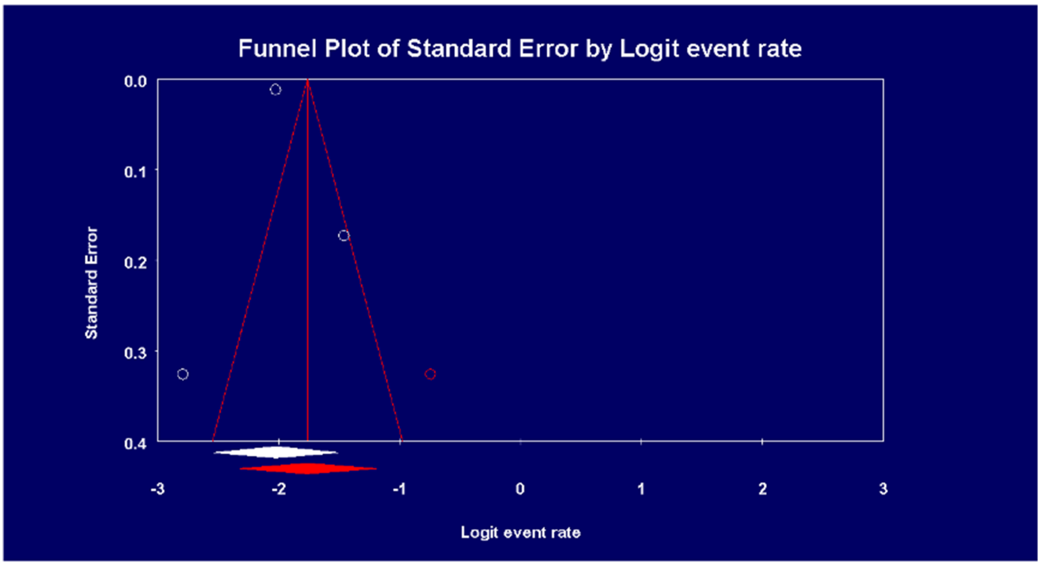

(a)

Classic fail-safe N

Z-value for observed studies	-104.81728
P-value for observed studies	0.00000
Alpha	0.05000
Tails	2.00000
Z for alpha	1.95996
Number of observed studies	3.00000
Number of missing studies that would bring p-value to > alpha	8578.00000

(b)

Duval and Tweedie's trim and fill

		Fixed Effects			Random Effects			Q Value
	Studies Trimmed	Point Estimate	Lower Limit	Upper Limit	Point Estimate	Lower Limit	Upper Limit	
**Observed values**		0.11661	0.11415	0.11911	0.11704	0.07356	0.18120	16.28243
**Adjusted values**	1	0.11679	0.11434	0.11930	0.14661	0.08905	0.23190	31.69691

(c)

Figure S1. (**a**). Through publication bias analysis, a slight asymmetry in the funnel plot is seen for SE by Logit Mortality rate for frail and non-frail patients with SAH. The red circle represents “missing” studies that have theoretically never been published due to publication bias, and the red diamond represents the “true” Logit event rate This could indicate potential publication bias present in this meta-analysis as missing studies could alter the the mean pooled mortality rate. (**b**) By using deploying Duval and Tweedie’s trim and fill analysis, we

can hypothetically expect the mortality rate to increase from 11.7% to 14.7% if publication bias could be eliminated. However, this does not mean our original results are null. (**c**) By utilizing Classic fail-safe N analysis, we see that 8578 hypothetical missing studies would be required to raise the p-value > 0.05. That is over 2859 missing studies per study included in this meta-analysis, and therefore we believe our results (mortality rate of 11.7% CI 7.4% - 18.1%, $p < 0.001$) are not null. In small meta-analyses (< 7 studies), the point I2 statistic can overestimate heterogeniety. Although I2 was less than 50 (I2 = 46.9), results should still be interpreted caution and with potential heterogeneity in mind because Q > Q (df). Regardless, CI was reported in place of I2 as suggested by von Hippel et al [30].
